# Estimates of SARS-CoV-2 Omicron BA.2 Subvariant Severity in New England

**DOI:** 10.1001/jamanetworkopen.2022.38354

**Published:** 2022-10-25

**Authors:** Zachary H. Strasser, Noah Greifer, Aboozar Hadavand, Shawn N. Murphy, Hossein Estiri

**Affiliations:** 1 MGH Laboratory of Computer Science, Massachusetts General Hospital, Boston; 2 Department of Medicine, Massachusetts General Hospital, Harvard Medical School, Boston, Massachusetts; 3 Institute for Quantitative Social Science, Harvard University, Cambridge, Massachusetts; 4 College of Computational Science, Minerva University, San Francisco, California; 5 Department of Neurology, Massachusetts General Hospital, Harvard Medical School, Boston

## Abstract

**Importance:**

The SARS-CoV-2 Omicron subvariant, BA.2, may be less severe than previous variants; however, confounding factors make interpreting the intrinsic severity challenging.

**Objective:**

To compare the adjusted risks of mortality, hospitalization, intensive care unit admission, and invasive ventilation between the BA.2 subvariant and the Omicron and Delta variants, after accounting for multiple confounders.

**Design, Setting, and Participants:**

This was a retrospective cohort study that applied an entropy balancing approach. Patients in a multicenter inpatient and outpatient system in New England with COVID-19 between March 3, 2020, and June 20, 2022, were identified.

**Exposures:**

Cases were assigned as being exposed to the Delta (B.1.617.2) variant, the Omicron (B.1.1.529) variant, or the Omicron BA.2 lineage subvariants.

**Main Outcomes and Measures:**

The primary study outcome planned before analysis was risk of 30-day mortality. Secondary outcomes included the risks of hospitalization, invasive ventilation, and intensive care unit admissions.

**Results:**

Of 102 315 confirmed COVID-19 cases (mean [SD] age, 44.2 [21.6] years; 63 482 women [62.0%]), 20 770 were labeled as Delta variants, 52 605 were labeled as the Omicron B.1.1.529 variant, and 28 940 were labeled as Omicron BA.2 subvariants. Patient cases were excluded if they occurred outside the prespecified temporal windows associated with the variants or had minimal longitudinal data in the Mass General Brigham system before COVID-19. Mortality rates were 0.7% for Delta (B.1.617.2), 0.4% for Omicron (B.1.1.529), and 0.3% for Omicron (BA.2). The adjusted odds ratio of mortality from the Delta variant compared with the Omicron BA.2 subvariants was 2.07 (95% CI, 1.04-4.10) and that of the original Omicron variant compared with the Omicron BA.2 subvariant was 2.20 (95% CI, 1.56-3.11). For all outcomes, the Omicron BA.2 subvariants were significantly less severe than that of the Omicron and Delta variants.

**Conclusions and Relevance:**

In this cohort study, after having accounted for a variety of confounding factors associated with SARS-CoV-2 outcomes, the Omicron BA.2 subvariant was found to be intrinsically less severe than both the Delta and Omicron variants. With respect to these variants, the severity profile of SARS-CoV-2 appears to be diminishing after taking into account various factors including therapeutics, vaccinations, and prior infections.

## Key Points

**Question:**

Is the SARS-CoV-2 Omicron BA.2 subvariant lineage intrinsically less severe than previous variants after taking into account demographics, comorbidities, prior infections, vaccinations, and treatments?

**Findings:**

In this cohort study of 101 470 patients, mortality rates were 0.7% for Delta (B.1.617.2), 0.4% for Omicron (B.1.1.529), and 0.3% for Omicron (BA.2) subvariants. After adjusting for confounding factors, the risk of death was significantly lower with the Omicron subvariant BA.2 compared with those of the Omicron and Delta variants.

**Meaning:**

Observational data that adjusts for confounders suggest that the Omicron BA.2 variant is intrinsically less severe than previous variants.

## Teaser

This cohort study evaluates the odds of mortality in patients infected with the SARS-CoV-2 Omicron BA.2 subvariant compared with the Delta (B.1.617.2) and Omicron (B.1.1.529) variants in New England.

## Introduction

The B.1.1.529 (Omicron) variant of SARS-CoV-2 has been reported as more transmissible, but less severe, than previous variants in a variety of locations including South Africa, Scotland, England, and Canada.^1,2,3,4^ Subsequently, other Omicron subvariants have become dominant in the population.^5^ Quantifying the intrinsic severity of these new subvariants remains challenging.^6^ Some cohort studies have attempted to quantify the severity of the Omicron BA.2 lineages but have shown different outcomes.^7,8^ This retrospective cohort study is the largest published that we know of to examine the severity of the BA.2 lineage and accounts for a variety of likely confounding factors.

Many factors have changed throughout the course of the pandemic that affect COVID-19 outcomes. This includes variations in the vulnerability of those infected,^9^ implementation of various public health strategies,^10^ new vaccines and therapeutics,^11,12,13,14^ and changes in testing.^15,16^ The increasing use of at-home antigen tests^17^ likely impacts the ability to evaluate the real severity of the new variants as fewer cases are recorded using polymerase chain reaction (PCR) tests in health care networks. Any comparison between SARS-CoV-2 variants without adequately adjusting and controlling for important confounders that may change over time can mislead both the public and medical experts of the true danger of the variant. It could also lead to mistrust among the public and poor choices by health policy experts.

To reduce confounding biases for understanding the intrinsic severity of the Omicron BA.2 subvariant compared with the Delta variant and the Omicron (B.1.1.529) variant, we leveraged Mass General Brigham’s (MGB) state-of-the-art electronic health record (EHR) system linked to the MGB COVID-19 enclave vaccine registry. The vaccine registry included vaccination records from multiple sources, including the state-wide Massachusetts Immunization Information System, the MGB COVID-19 vaccine administration, and the Epic documentation of a vaccination according to a patient’s report of a COVID-19 vaccination to their physician. The longitudinal records include patient demographics, comorbidities, and previous SARS-CoV-2 infections. Additionally, if a patient had a positive antigen test at home and spoke with an MGB clinician over the telephone, their EHR record was flagged as having COVID-19 despite not having a positive PCR test in the system. Leveraging this very expansive EHR system and the Massachusetts vaccination record resource allows for the development of a model that can more accurately account for possible confounding covariates. Then entropy balancing was applied to compare the Omicron subvariant with the Delta and Omicron variants.

## Methods

The use of data was approved by the MGB institutional review board protocol, with a specified waiver of consent for a data-only retrospective study. MGB is an integrated health care delivery system with 14 hospitals and hundreds of local private clinics and health care centers serving 1.5 million patients annually in the New England region. Our study followed the Strengthening the Reporting of Observational Studies in Epidemiology (STROBE) reporting guideline for cohort studies.

### Disease Cohort Assembly

Adult and child COVID-19 cases were identified between March 3, 2020, and May 21, 2022. Patients were identified as having a positive test by 1 of 2 methods. They could have a positive PCR (see eTable 1 in the Supplement for a list of positive results included), or they were identified with a confirmed flag or presumed flag in the EHR. SARS-CoV-2 PCRs within the MGB system automatically received a confirmed flag. However, if a PCR was outside of the MGB system but still within Epic’s EHR, it would also be labeled a confirmed flag. Finally, if a patient had an at-home antigen test and called their clinician, the clinician could identify this patient as having either a confirmed or presumed COVID-19 flag. The COVID-19 diagnosis codes were not used because previous internal reviews had shown them to be less accurate than the PCR test and EHR flags. For example, diagnostic codes could refer to the incidental date of infection, but they also may refer to a follow-up appointment.^18^

Cases were categorized as being part of 1 of 3 groups (Delta, Omicron, or Omicron subvariant) according to the timing of the onset of disease detection. Period barriers were determined by variant dominance on residual diagnostic tests sent to the Broad Institute in Boston, Massachusetts, and the US Centers for Disease Control and Prevention’s COVID-19 tracking system.^5,19^ Cases between June 27, 2021, and December 5, 2021, were classified as Delta. A buffer period when Omicron and Delta variants were detected between December 6, 2021, and December 25, 2021, was excluded. Cases between December 26, 2021, and February 21, 2022, were classified as Omicron. Another buffer period was used between February 22 and March 20, 2022. Then any cases between March 21 and May 21, 2022 were classified as the Omicron BA.2 subvariant.

The first positive PCR or flag for a given patient was considered the incident date of the disease. If a patient had another positive PCR or new flag greater than 90 days from the incident date, this was considered to be a new case of COVID-19.^20,21^

To increase the likelihood that there was enough information for a patient to compute their comorbidity history and capture their outcomes in follow-up care with the MGB system clinicians, a minimum data floor threshold of at least 2 diagnosis records, 6 months apart, in the 3 years before the SARS-CoV-2 infection was required.

### Covariates

Baseline characteristics were collected from the EHR. The Elixhauser comorbidity index score was calculated in R by extracting *International Statistical Classification of Diseases and Related Health Problems, Tenth Revision* codes and using the R package comorbidity to then calculate the Elixhauser scores for each patient at the time of diagnosis.^22^ The Elixhauser comorbidity groups were also recorded. The comorbidities were then reorganized into distinct classes (see eAppendix in the Supplement).

The vaccine enclave, which included the state-wide Massachusetts Immunization Information System, the MGB COVID-19 vaccine administration data, and the Epic documentation of a vaccination according to a patient’s report of a COVID-19 vaccination to their physician, were linked to the local MGB data repository. The vaccination status for each patient 21 days before the COVID-19 incident date was categorized into 1 of 4 groups: no vaccine, first dose only (representing the first dose of Moderna or Pfizer BioNTech), fully vaccinated (representing the second dose of Moderna or Pfizer BioNTech or Johnson and Johnson), or fully vaccinated with booster (representing any of the vaccines with an additional booster given).

Race and ethnicity were used as covariates in this study because COVID-19 deaths have occurred disproportionately among certain racial and ethnic minority groups.^23^ The racial category was chosen from 1 of 4 categories including White, Black or African American, Asian, or Other/Unknown. Other includes self-identified categories of Native Hawaiian or other Pacific Islander, American Indian or Alaskan Native, Arab, multirace, or other. Unknown refers to unknown or declined to answer. Ethnicity was categorized as Hispanic or not. Both race and ethnicity were self-identified by patients within their EHR.

Cases were labeled as being reinfections according to whether the COVID-19 test was positive again greater than 90 days from the previous infection incident date. Patients were also classified as having received steroids or antivirals since these have been found in randomized clinical trials to be some of most effective treatments for decreasing COVID-19 severity. Antivirals included any prescription for nirmatrelvir-ritonavir or remdesivir within 30 days of the incident date. Steroids included a prescription of dexamethasone within 30 days of the incident date.

### Outcomes

The primary outcome was defined as an in-hospital death within 30 days of the positive COVID-19 case. Secondary outcomes included hospitalization, invasive ventilation, and intensive care unit admissions. If these events occurred within 30 days from the COVID-19 incident date, the patient was designated as having one of the secondary outcomes.

### Sample Size Calculation

Given the descriptive nature of this study, sample size calculations were not computed. The cohort size was fixed by the chosen criteria. Adequacy of the sample size was assessed by the 95% CIs around the primary point estimates.

### Statistical Analysis

First, Fisher exact test was used to calculate the unadjusted odds ratios (ORs) to compare the Omicron BA.2 subvariant outcomes with the Delta and Omicron variant outcomes. A statistically significant level of two-tailed *P* < .05 was used. Then to reduce the confounding bias, cases were classified according to the patient’s age, Elixhauser comorbidity index score, vaccination status, ethnicity and race, sex, and previous infection status. Entropy balancing, a type of inverse probability weighting, was used to estimate the average treatment effect size in the population, which captures the average differences in the outcomes due to the variants across the full population represented by the sample.^24^ Entropy balancing guarantees exact balance on the covariate means while increasing precision over traditional inverse probability weights. All covariates were included in estimating the entropy balancing weights. The primary focus was the OR of the outcomes between the Omicron BA.2 subvariant and the Delta and Omicron variants. The treatment effect sizes and the corresponding 95% CIs were estimated using a weighted logistic regression model with robust (sandwich) SEs. No covariates were included in the outcome model. R statistical software version 3.6.3 (R Project for Statistical Computing) was used for analysis. The R package Weightit was used for estimating the entropy balancing weights,^25^ the R package cobalt was used for checking balance,^26^ and the R package survey was used to estimate the weighted ORs and 95% CIs.^27^ The code for analysis is available online.^28^

## Results

**Figure 1.**
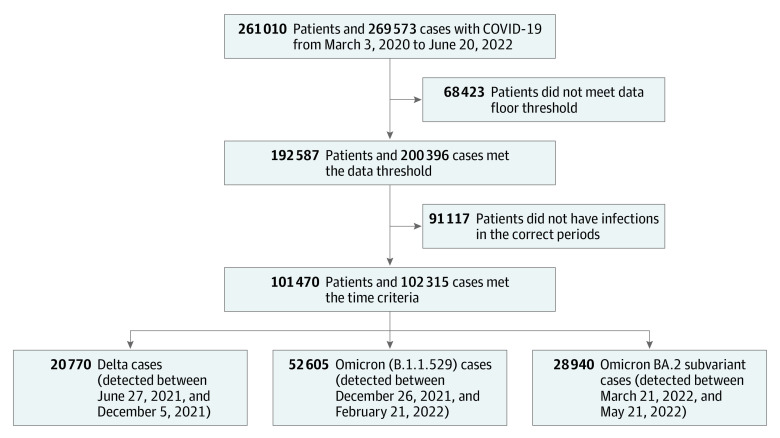
Identification of Omicron and Delta Cases in Mass General Brigham Hospital

From March 3, 2020, and June 20, 2022, 102 315 unique SARS-CoV-2 cases met the inclusion and exclusion criteria for the study (Figure 1), which included 63 482 women (62.0%) and an overall mean (SD) age of 44.2 (21.6) years. The majority of patients, 78 133 (76.4%), self-identified as non-Hispanic White. Of these total cases, 20 770 cases were labeled as the Delta variant, 52 605 cases were labeled as the Omicron variant, and 28 940 were labeled as the Omicron BA.2 subvariant. Table 1 summarizes the key characteristics for the population that contracted each of the 3 variants.

**Table 1.** Baseline Characteristics of Study Cohort

Characteristics	Patients, No. (%)		
	Delta (n = 20 770)	Omicron (n = 52 605)	Omicron subvariant (n = 28 940)
Sex			
Male	8675 (41.8)	19 606 (37.3)	10 552 (36.5)
Female	12 095 (58.2)	32 999 (62.7)	18 388 (63.5)
Age, y			
Mean (SD)	43.4 (22.1)	41.6 (21.5)	49.5 (20.5)
Median (IQR)	44 (27-60)	41 (26-58)	51 (35-64)
Race and ethnicity			
Hispanic	4 (0.02)	18 (3.4)	3 (0.01)
Non-Hispanic			
Asian	476 (2.3)	2036 (3.9)	1370 (4.7)
Black	1349 (6.5)	4299 (8.2)	1072 (3.7)
White	16 472 (79.3)	37 689 (71.6)	23 972 (82.8)
Other or unknown^a^	2469 (11.9)	8563 (16.3)	2523 (8.7)
Comorbidities			
High blood pressure	4647 (22.4)	11 442 (21.8)	8132 (28.1)
Immunosuppressed	2177 (10.5)	5898 (11.2)	4452 (15.4)
Diabetes	1824 (8.8)	4694 (8.9)	2708 (9.4)
Chronic pulmonary disease	2829 (13.6)	7877 (15.0)	4529 (15.6)
Depression	2979 (14.3)	7877 (15.0)	4529 (15.6)
Liver disease	993 (4.8)	2808 (5.3)	1646 (5.7)
Pulmonary circulation disease	290 (1.4)	846 (1.6)	544 (1.9)
Vaccine			
None	9292 (44.7)	12 791 (24.3)	2841 (9.8)
First dose	861 (4.1)	2397 (4.6)	1009 (3.5)
Fully vaccinated	10 081 (48.6)	22 442 (42.6)	6016 (20.8)
Fully vaccinated with booster	536 (2.6)	14 975 (28.5)	19 074 (65.9
Prior infection	378 (1.8)	3350 (6.4)	1532 (5.3)
Medications during acute illness			
Nirmatrelvir-ritonavir	0	105 (0.2)	7702 (26.6)
Remdesivir	801 (3.9)	1124 (2.1)	580 (2.0)
Steroids	1351 (6.5)	2045 (3.9)	1097 (3.8)

^a^ Other includes self-identified categories of Native Hawaiian or other Pacific Islander, American Indian or Alaskan Native, Arab, multi-race, or other. Unknown refers to unknown or declined to answer.

There were slight variations in demographics between each of the groups. The percentage of men identified with SARS-CoV-2 was highest with the Delta variant (8675 men [41.8%]) and lowest with the Omicron subvariant (19 606 men [37.3%]). Age was slightly higher for the Omicron subvariant with a mean (SD) age of 49.5 (20.5) years and median (IQR) of 51 (35-64) years. The majority of patients among the Delta variant were non-Hispanic White (16 472 [79.3%]); however, this varied from 37 689 (71.6%) cases with the Omicron variant to 23 972 (82.8%) with the Omicron BA.2 subvariant. The reported comorbidities were similar among all 3 populations. Notably, many cases were in patients with chronic pulmonary disease, including 2829 patients (13.6%) with the Delta variant, 7877 patients (15.0%) with the Omicron variant, and 4529 patients (15.6%) with the Omicron BA.2 variant. The number of vaccinated patients changed substantially between variants. With the Delta variant, there was nearly an even split between fully vaccinated (10 081 patients [48.5%]) and unvaccinated (9292 patients [44.7%]) patients. As the pandemic progressed, most of the patients infected were vaccinated. During the Omicron subvariant’s surge, 19 074 patients (65.9%) were vaccinated with a booster. Additionally, prior infections were the lowest during the Delta wave at 378 patients (1.8%) and then increased to 3350 patients (6.4%) and 1532 patients (5.3%) during the Omicron and Omicron subvariant waves, respectively. Finally, treatments changed throughout the pandemic. Nirmatrelvir-ritonavir (Paxlovid) became available during the Omicron wave, and the number of prescriptions began to increase. Remdesivir and steroid prescription usage decreased during the pandemic.

The entropy balancing resulted in all variance ratios as being close to 1, all Kolmogorov-Smirnov statistics as being low (<0.03), and standardized mean differences of squares and cubes of the covariates as being less than 0.05 (see eTable 2 in the Supplement).

### Primary Outcome: 30-Day Mortality

According to the initial, raw end points, there were 148 recorded deaths within 30 days of infection with the Delta variant, whereas there were 203 recorded deaths with the Omicron variant and 76 with the Omicron BA.2 subvariant. This represented 0.7% of the detected Delta cases, 0.4% of the detected Omicron cases, and 0.3% of the Omicron BA.2 subvariant cases (Table 2).

**Table 2.** Unadjusted Outcomes of Delta and Omicron Variant COVID-19 Cases

Outcome	Patients, No. (%)		
	Delta variant (n = 20 770)	Omicron variant (n = 52 605)	Omicron subvariant (n = 28 940)
Hospitalization	1223 (5.9)	1720 (3.3)	911 (3.1)
Ventilation	158 (0.8)	232 (0.4)	102 (0.4)
Intensive care unit admission	203 (1.0)	272 (0.5)	103 (0.4)
Death^a^	148 (0.7)	203 (0.4)	76 (0.3)

^a^ Denotes in-hospital, 30-day mortality.

**Figure 2.**
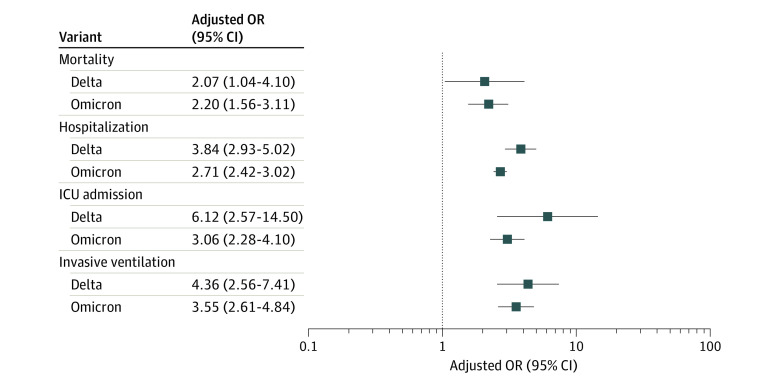
Adjusted Odds Ratios (ORs) After Application of the Inverse of Probability of Treatment Weighting The plot shows the odds of severe outcomes among patients infected with the Delta (B.1.617.2) or Omicron (B.1.1.529) variants vs the Omicron BA.2 subvariant (reference). ICU indicates intensive care unit.

After adjusting for the covariates including sex, age, race and ethnicity, comorbidities, vaccine status, treatments, and prior infection using entropy balancing, the weighted ORs show that Delta mortality was greater compared with Omicron subvariant mortality (OR, 2.07; 95% CI, 1.04-4.10) and the Omicron variant mortality was greater compared with Omicron subvariant mortality (OR, 2.20; 95% CI, 1.56-3.11) (Figure 2 and eTable 3 in the Supplement).

### Secondary Outcomes: Hospitalization, Invasive Ventilation, and Intensive Care Unit Admission

After adjustment, the risk of hospitalization with Delta was significantly higher than that of the Omicron subvariant (OR, 3.84; 95% CI, 2.93-5.02). Additionally, the Omicron variant was also much more likely to lead to hospitalization than the subvariant (OR, 2.71; 95% CI, 2.42-3.02).

An intensive care unit admission was much more likely with Delta than compared with the Omicron subvariant (OR, 6.12; 95% CI, 2.57-14.5) and with the Omicron variant compared with the subvariant (OR, 3.06; 95% CI, 2.28-4.10). Finally, invasive ventilation was also significantly more likely with Delta compared with the Omicron subvariant (OR, 4.36; 95% CI, 2.56-7.41) and with Omicron compared with the Omicron subvariant (OR, 3.55; 95% CI, 2.61-4.84).

## Discussion

In a community-based cohort of patients with COVID-19, significant differences in 30-day mortality, as well as hospital and intensive care unit admission and need for mechanical ventilation, were found between the Omicron subvariant BA.2 and that of Omicron and Delta variants after adjusting for covariates. The results suggest that the BA.2 lineage has become intrinsically less severe than that of the original Omicron variant and the Delta variant. The use of entropy balancing to equate the patient populations on a long list of background characteristics suggests that the observed differences in severity are not due to these characteristics but may instead reflect intrinsic differences in severity among the variants.

In vitro data previously suggested that Omicron has suboptimal cell fusion when compared with other variants.^29^ The fusion is thought to happen through a transmembrane serine protease 2-dependent process. The spread of the virus in the lungs depends to greater extent on the cell fusion protein; therefore, Omicron may be less likely than other variants to cause severe lung disease.^30^ Additionally, Syrian hamsters and human angiotensin-converting enzyme 2-expressing mice have been found to have milder lung disease with the Omicron infection.^31,32^ A number of general population studies have also shown that Omicron appears to lead to milder cases in the population.^1,2,3,4^

However, these studies have been limited to investigating the original Omicron variant (B.1.1.529), and not the BA.2 lineage. Limited data, both in vitro and clinically, exist on the BA.2 lineage. In a limited population of 207 BA.2 cases by Gautret et al,^8^ only 3 very elderly patients died, suggesting potentially reduced severity compared with the Omicron B.1.1.529 variant. Lewnard et al^7^ examined a larger cohort of BA.2-infected individuals, but did not find a significant difference in risk between the subvariant and the B.1.1.529 lineages. However, their study only included 1905 cases of the subvariant, compared with 28 940 cases of the subvariant in our study. Additionally, we identified a greater number of patients with reinfection including 6.4% in the Omicron era, than in the study by Lewnard et al,^7^ suggesting that our study may have been better able to account for reinfections, given how we defined this in the EHR.

Additionally, the use of entropy balancing has several advantages over other matching methods. For instance, compared with other propensity score–matching techniques, unmatched individuals are not discarded when using entropy balancing, which can increase the precision of the effect size estimates and the generalizability of the findings.^33^

To our knowledge, this study is the most comprehensive for taking into account various substantial confounders likely associated with the outcomes of patients with COVID-19. In addition, this study accounts for home-based antigen tests by using EHR flags that designate a SARS-2-CoV infection. As antigen tests have become more prevalent, factoring in the clinician flag in the EHR can lead to capturing the majority of patients who may not take a PCR test. This study also linked state-level vaccine records to a comprehensive EHR system that accounts for patient comorbidities and complex outcomes. This more extensive covariate modeling allows for better adjustments of covariates and a more accurate measurement of the intrinsic nature of the infection.

### Limitations

This study has limitations. Despite including flagged COVID-19–positive patients, unobserved infections are still a potential source of bias when comparing severe outcomes. Only 1 in 2.5 infections in the state of California were estimated to have been caught by testing.^34^ This could lead to underestimating the overall count and not fully accounting for the reinfection rate. To help reduce this limitation, we allowed the use of COVID-19 flags in the EHR, to identify the tests performed at home but not documented in the EHR through a PCR. Additionally, although the vaccination records included 3 separate workflows, including the Massachusetts Immunization Information System, it may have still missed some patients’ vaccinations, thereby underestimating vaccinated patients and overestimating unvaccinated patients. The cohort only included patients with a minimal data floor threshold. Although this increases the likelihood they sought care within the MGB health care system, it likely leads to studying a population with more comorbidities than the general public. However, since we are comparing different variants, they each should have been affected similarly and have a similar population for comparison. As with all observational studies, the possibility of bias due to remaining unmeasured confounding remains. This includes missingness that may exist in patients’ medical records. Additionally, the primary outcome is an in-hospital death within 30 days. We likely underestimated the number of deaths by only including deaths within the specified window and within the hospital. Still, we expect that these general limitations of observational studies would have been evenly distributed between all variants and would not have had a significant adverse effect on the results.

## Conclusions

Measuring the intrinsic severity of the new Omicron subvariant can be challenging given the many confounders that have changed throughout the pandemic. Combining the EHR of a large, integrated health care system with state-level vaccination records can provide a more accurate understanding of how variants compare with one another.

## Notes

Supplement. **eTable 1. **Positive Results Accepted for a COVID-19 PCR Test **eTable 2. **Balance Summary Across all Treatment Pairs **eTable 3. **Unadjusted and Adjusted Odds Ratios Comparing Omicron Subvariant to Various Variants **eAppendix. **Chronic Condition Indicators
