# Supplementary material for: Estimates of SARS-CoV-2 Omicron BA.2 Subvariant Severity in New England
Source: JAMA Netw Open. 2022 Oct 25;5(10):e2238354. doi: 10.1001/jamanetworkopen.2022.38354 (PMC9597387; doi:10.1001/jamanetworkopen.2022.38354)
Supplement: Supplement. — eTable 1. Positive Results Accepted for a COVID-19 PCR Test eTable 2. Balance Summary Across all Treatment Pairs eTable 3. Unadjusted and Adjusted Odds Ratios Comparing Omicron Subvariant to Various Variants eAppendix. Chronic Condition Indicators [file jamanetwopen-e2238354-s001.pdf]

Supplemental Online Content

Strasser ZH, Greifer N, Hadavand A, Murphy SN, Estiri H. Estimates of SARS-CoV-2 Omicron BA.2 subvariant severity in New England. *JAMA Netw Open*. 2022;5(10):e2238354. doi:10.1001/jamanetworkopen.2022.38354

eTable 1. Positive Results Accepted for a COVID-19 PCR Test
eTable 2. Balance Summary Across All Treatment Pairs
eTable 3. Unadjusted and Adjusted Odds Ratios Comparing Omicron Subvariant to Various Variants
eAppendix. Chronic Condition Indicators

This supplemental material has been provided by the authors to give readers additional information about their work.

© 2022 Strasser ZH et al. *JAMA Network Open.*

eTable 1: Positive Results Accepted for a COVID PCR Test

Positive results accepted for a COVID PCR test
SARS-COV-2 DETECTED
DETECTED
Positive
SARS-CoV-2 detected
Detected
Positive for 2019-novel Coronavirus (2019-nCoV) by PCR
POSITIVE
POSITIVE FOR 2019-NOVEL CORONAVIRUS (2019-NCOV) BY PCR.
PRESUMPTIVE POSITIVE
Presumptive Positive
POSITIVE FOR SARS-COV-2 (COVID-19) BY PCR
POSITIVE (Detected)
Detected by PCR
positive
postive
Covid-19 Positive

© 2022 Strasser ZH et al. *JAMA Network Open.*

detected
Positive for SARS-CoV02 (2019 novel coronavirus)by isothermal nucleic acid amplification technology. This test has been authorized by the FDA under an Emergency Use Authorization (EUA) for use by authorized laboratories.
SARS-CoV-2
GROUP COVID POSITIVE
POSITIVE results re indicative of active infection with SARS-CoV-2. Clinical correlation is necessary to determine patient infection status.
THE SPECIMEN IS PRESUMPTIVELY POSITIVE FOR SARS-COV-2, THE CORONAVIRUS ASSOCIATED WITH COVID-19. THIS RESULT WAS UNABLE TO BE CONFIRMED AS POSITIVE BY A SECOND TEST METHOD
POSITIVE-Valid Control
positve
Positive for COVID19 (SARS CoV2) by PCR
pos
POSITIVE
SARS-CoV-2 (Agent of COVID-19) Detected by PCR.
postivie
PRESUMPTIVE POSITIVE FOR SARS-COV-2 (COVID-19) BY PCR
COVID DETECTED
Presumptive positive for SARS-Cov-2 (COVID-19) by PCR

© 2022 Strasser ZH et al. *JAMA Network Open.*

DETECTED SARS-CoV-2 (COVID-19)
Positive SARS-CoV-2
DETECTED!
SARS-CoV-2 Detected
Positive for 2019-nCoV
positive for 2019-nCOV!
COVID 2 DAY Positive
POSITIVE SARS-COV-2
POSITIVE
COVID-19 Positive
Positive/Detected
Detected!

© 2022 Strasser ZH et al. *JAMA Network Open.*

eTable 2: Balance Summary Across all Treatment Pairs

Variable Label	Definition	Type	SMD*	Variance Ratio	KS Statistic
vaccine_status_0	No vaccine	Binary	0	.	0
vaccine_status_1	1 dose of the vaccine	Binary	0	.	0
vaccine_status_2	2 doses of the vaccine	Binary	0	.	0
vaccine_status_3	Fully vaccine and Booster	Binary	0	.	0
hispanic	Hispanice ethnicity	Binary	0	.	0
race_0WHITE	White	Binary	0	.	0
race_ASIAN	Asian	Binary	0	.	0
race_BLACK	Black	Binary	0	.	0
race_Other/Unknown	Unknown	Binary	0	.	0
age	Age	Contin.	0	1.126	0.035
female	Sex	Binary	0	.	0
elixhauser_index	Elixhauser Comorbidity Index	Contin.	0	1.094	0.012
prior_infection	Number of Prior Infections	Contin.	0	1.095	0.002

© 2022 Strasser ZH et al. *JAMA Network Open.*

anti.viral	Anti-viral medications	Binary	0	.	0
Steroids	Steroids provided	Binary	0	.	0
age^2		Contin.	0.029	1.076	0.035
$elixhauser\_index^2$		Contin.	0.023	1.229	0.012
$prior\_infection^2$		Contin.	0.02	1.562	0.002
age^3		Contin.	0.038	1.118	0.035
$elixhauser\_index^3$		Contin.	0.034	1.293	0.012
$prior\_infection^3$		Contin.	0.045	2.608	0.002

* Balanced, <0.05

SMD = standardized mean difference; KS = Kolmogorov-Smirnov

All statistics represent the largest observed value across all pairwise comparisons among variants.

© 2022 Strasser ZH et al. *JAMA Network Open.*

eTable 3: Unadjusted and Adjusted Odds Ratios Comparing Omicron Subvariant to Various Variants

	Delta	Delta Adjusted	Omicron	Omicron Adjusted
Death	2.72 [2.06 – 3.59]	2.07 [1.04 – 4.10]	1.47 [1.13 – 1.92]	2.20 [1.56 – 3.11]
Hospitalization	1.93 [1.76 – 2.10]	3.84 [2.93 – 5.02]	1.04 [0.96 – 1.13]	2.71 [2.42 – 3.02]
Ventilation	2.17 [1.69 – 2.78]	4.36 [2.56 – 7.41]	1.25 [0.99 – 1.58]	3.55 [2.61 – 4.84]
ICU Admission	2.76 [2.18 – 3.51]	6.12 [2.57 - 14.5]	1.46 [1.16 – 1.83]	3.06 [2.28 – 4.10]

eAppendix

Chronic condition indicators- Each chronic condition indicator was set equal to 1 if the person had at least one of a list of ICD-10-CM diagnosis codes in the electronic health record from up to 14 days before the diagnosis.
The following groupings were used:
Hypertension (hypunc and hypc),
Comorbidities with immunosuppression (aids, rheumd, lymph, metacanc, solidtum), Diabetes (diabunc and diabc),
Chronic pulm disease (cpd),
Depression (depre),
LIver disease (ld),
Pulmonary circulation disorders (pcd)

© 2022 Strasser ZH et al. *JAMA Network Open.*
